# Brain-Derived Neurotropic Factor* Val66Met* Polymorphism and Posttraumatic Stress Disorder among Survivors of the 1998 Dongting Lake Flood in China

**DOI:** 10.1155/2017/4569698

**Published:** 2017-05-14

**Authors:** Wenjie Dai, Atipatsa C. Kaminga, Xin Wu, Shi Wu Wen, Hongzhuan Tan, Junxia Yan, Jing Deng, Zhiwei Lai, Aizhong Liu

**Affiliations:** ^1^Department of Epidemiology and Health Statistics, Xiangya School of Public Health, Central South University, Changsha, Hunan 410008, China; ^2^Department of Mathematics, Mzuzu University, Private Bag 201, Mzuzu 2, Malawi; ^3^OMNI Research Group, Department of Obstetrics and Gynecology, Faculty of Medicine, University of Ottawa, Ottawa, ON, Canada; ^4^Ottawa Hospital Research Institute, Clinical Epidemiology Program, Ottawa, ON, Canada; ^5^School of Epidemiology, Public Health, and Preventive Medicine, Faculty of Medicine, University of Ottawa, Ottawa, ON, Canada; ^6^Immune Planning Division, Hunan Provincial Center for Disease Control and Prevention, Changsha, Hunan 410005, China

## Abstract

**Objective:**

This study mainly aimed to explore the association between brain-derived neurotropic factor (BDNF)* Val66Met* polymorphism and posttraumatic stress disorder (PTSD) among flood survivors in China.

**Methods:**

Individuals who experienced the 1998 Dongting Lake flood in Southeast Huarong, China, were enrolled in this study. Qualified health personnel carried out face-to-face interviews with participants. PTSD was identified using PTSD Checklist-Civilian version (PCL-C). Blood samples were collected from the participants to extract DNA for genotyping.

**Results:**

A total of 175 participants were enrolled in this study. The prevalence of PTSD among flood survivors at 17-year follow-up was 16.0% (28/175). Individuals with PTSD were more likely to be female, experience at least three flood-related stressors, experience at least three postflood stressors, and carry the* Met* than those without PTSD. Compared with* Val/Val* homozygotes,* Met* carriers had higher scores of PCL-C (mean ± standard error: 23.60 ± 7.23 versus 27.19 ± 9.48, *P* < 0.05). Multivariable logistic regression analysis indicated that* Met* carriers (aOR = 4.76, 95% CI = 1.02–22.15, *P* < 0.05) were more likely to develop PTSD than* Val/Val* homozygotes.

**Conclusions:**

* Met* carriers for BDNF rs6265 are at higher risk of developing PTSD and also exhibit more severe PTSD symptoms than* Val/Val* homozygotes among flood survivors in China.

## 1. Introduction

Flood is one of the most common and devastating natural disasters. For example, according to the official statistics, a flood which struck South Korea in 2002 destroyed 17,000 houses and other forms of property worth $6.6 billion [1] and a flood which struck England in 2007 caused 13 deaths and evacuation of 7,000 people [2]. In China, a flash flood in the Yangzi River in 1998, which hit hard the Dongting Lake area, affected over 180 million people, damaged 6.85 million houses, left 18.393 million people homeless, and caused 4,150 deaths and an economic loss of $32 billion [3]. Floods can lead to not only economic loss and physical injuries, but also long-term mental health problems [4, 5].

Posttraumatic stress disorder (PTSD) is the most prevalent psychiatric disorder among flood survivors [6], with the reported prevalence ranging from 8.6% to 43.1% [7, 8]. In general, there has been four major PTSD trajectories following traumatic events: (I) resilient or resistant status with consistently quite slight PTSD symptoms, (II) recovery from distress then remission over time, (III) delayed onset with worsening PTSD symptoms over time, and (IV) chronic status with consistently severe PTSD symptoms [9, 10]. For example, Bryant et al. examined long-term PTSD trajectories over 6 years in 1,084 randomly selected injury patients and found five PTSD trajectories across the 6 years using five waves of assessments (baseline, 3 months, 12 months, 24 months, and 6 years). Specifically, 73% were resilient, 6% were recovery, 10% were worsening, 4% were chronic, and 8% were worsening and then recovered [11].

In addition to the prevalence over time, genetic background has been shown to play an important role in the onset and course of PTSD [12–14]. The brain-derived neurotropic factor (BDNF) gene, a member of the nerve growth factor family of neurotrophins, plays an important role in the regulation, differentiation, and maintenance of neuronal populations in the peripheral and central nervous systems [15]. BDNF has been implicated in various psychiatric disorders, such as bipolar disorder, schizophrenia, and anxiety [16–18]. Moreover, since BDNF plays an irreplaceable role in stress reaction and memory [19, 20], which is associated with some clinical manifestations of PTSD, the role of BDNF in PTSD has also been widely explored. Su et al. found that higher BDNF plasma levels may be a protective factor for trauma-related subjects from developing PTSD [21]. Stratta et al. found that PTSD patients showed significantly lower plasma levels of BNDF [22]. Furthermore, Hauck et al. found that BDNF levels were not stable in traumatized groups over time [23], as a consequence of which PTSD trajectory may be affected.

BDNF* Val66Met* (also known as rs6265, G196A) polymorphism, which affects synaptic localization and plays a crucial role in activity-dependent secretion of BDNF from the neurons [24], is a functional single nucleotide polymorphism (SNP) of the BDNF gene. Studies in war-related populations found that psychotic veterans with PTSD carried more* Met* alleles than nonpsychotic veterans with PTSD or veterans without PTSD [25], and, in soldiers diagnosed with PTSD after their involvement in the Iraq and Afghanistan wars, the frequency of* Met/Met* genotype was almost threefold higher than that in the controls [26]. Furthermore, the fact that BDNF serum levels were lower in* Met* carriers than in* Val/Val* homozygotes [27, 28] indicated that BDNF* Val66Met* polymorphism may be involved in the dynamic alteration of BDNF levels, and, therefore, implicated in the clinical course of PTSD. This was supported by Li et al., who found that the association between BDNF Val66Met and PTSD varied longitudinally among Chinese adolescents following the 2008 Wenchuan earthquake [29].

However, studies focusing on populations exposed to different types of trauma have exhibited quite inconsistent results when exploring the association between BDNF* Val66Met* polymorphism and PTSD [26, 30–32], suggesting that the type of trauma exposure may affect this association [16, 33]. Up to now, there has been no study attempting to explore the association between BDNF* Val66Met* polymorphism and PTSD among flood survivors. Thus, in this study, PTSD cases among survivors of the 1998 Dongting Lake flood in China were identified to examine the association between BDNF* Val66Met* polymorphism and PTSD.

## 2. Materials and Methods

### 2.1. Ethics Approval

The investigation was performed in accordance with the latest version of the Declaration of Helsinki and approved by the Ethics Committee of Xiangya School of Public Health, Central South University of China. Written informed consent was obtained from the participants.

### 2.2. Participants

This cross-sectional study was conducted in the Southeast of Huarong County, which was seriously affected by the 1998 Dongting Lake flood. Huarong County, a catchment area of Dongting Lake, is located in the south of the middle reach of the Yangzi River, and the Southeast of Huarong County is directly next to Dongting Lake. Six villages were randomly selected from the southeast of Huarong County to form the sampling frame for this survey. These villages had common characteristics with all other villages in this area, in terms of the severity of the damage caused by the Dongting Lake flood in 1998. All eligible participants in these villages were enrolled in this study. The inclusion criteria comprised (1) being 7 years of age or older in 1998; (2) having experienced Dongting Lake flood in 1998; (3) willing to participate; and (4) willing to provide blood samples. In accordance with a previous study targeting the survivors of the same flood disaster [7], individuals aged at least 7 years were enrolled in this study since evidence has shown that flood may have psychological impact on not only adults (≥16 years) but also children or adolescents aged between 7 and 15 years [34, 35]. Excluded from this study were subjects who (1) could not communicate clearly, such as people with dementia or severe diseases; (2) have suffered from other kinds of psychiatric disorders, such as depression, schizophrenia, or anxiety; (3) have taken psychotropic drugs or received psychological intervention since the Dongting Lake flood in 1998.

### 2.3. Procedure

This study was conducted in December 2015, almost 17 years after the 1998 Dongting Lake flood. Data collection was done by qualified health personnel who were recruited from medical school graduates or experienced workers in the Huarong Center for Disease Control and Prevention. Prior to data collection, they were given a uniform rigorous training on data collection specific for this study. After the training, they carried out face-to-face interviews with participants using a questionnaire to collect data related to demographics, flood-related stressors, postflood stressors, and PTSD symptoms. Blood samples were collected from the participants to extract DNA and determine BDNF* Val66Met* genotypes.

### 2.4. Measurements

#### 2.4.1. Flood-Related and Postflood Stressors

Flood-related stressors were used to examine the degree of exposure to the traumatic event, the Dongting Lake flood in 1998. As reported previously in most studies on natural disasters [36, 37], flood-related stressors were identified by asking the following yes/no questions. Did you have your homes destroyed by the 1998 Dongting Lake flood? Did you or your family lose livelihood due to the 1998 Dongting Lake flood? Did you or your family lose most property due to the 1998 Dongting Lake flood? Did you or your family members suffer from physical injuries due to the 1998 Dongting Lake flood? Did you lose at least one family member due to the 1998 Dongting Lake flood? In this way, the total number of flood-related stressors was obtained for each participant.

Postflood stressors were used to assess the stressful situations experienced by the participants after the Dongting Lake flood in 1998 until December 2015. The following questions were asked to identify postflood stressors. Did you or your family members experience stressful events (e.g., traffic accidents, cancers, divorce, and relative lost) after the Dongting Lake flood in 1998 until present? If the answer was yes, participants were asked to specify stressful events they experienced during that time period. In this way, the total number of postflood stressors was obtained for each participant.

#### 2.4.2. PTSD

PTSD was identified using the PTSD Checklist-Civilian version (PCL-C). The PCL-C is a 17-item self-report questionnaire based on the DSM-IV. This instrument has been widely used when the formal clinical evaluation was not feasible [38]. Each of the 17 items has five possible integer scores on a scale, ranging from 1 (not at all) to 5 (extremely). Exactly one score is assigned to each item. Thus, the total score of the PCL-C ranges from 17 to 85. Participants with a score of 38 or greater are diagnosed as having PTSD [39, 40]. The PCL-C has high internal consistency [41] and the Chinese version of the PCL-C has sound validity and reliability [42].

#### 2.4.3. DNA Extraction and Genotyping

Peripheral blood sample of 3 to 5 mL was collected from each participant and stored in ethylenediaminetetraacetic acid (EDTA) tubes at −20°C until required. Genomic DNA was extracted from each of the peripheral blood samples using a TIANamp Blood DNA Kit (Tiangen Biotech, Beijing, China) according to the instructions. Genotyping was performed using the Sequenom MassARRAY iPLEX platform (Sequenom Inc., San Diego, California, USA) [43]. Specifically, automated genotyping involves the following 6 steps: (I) isolation, quantitation, and amplification of DNA; (II) preparation of iPLEX Gold reaction products; (III) transfer of iPLEX Gold reaction products to SpectroCHIPs; (IV) defining the setup of assays and plates in the MassARRAY database; (V) acquisition of spectra; and (VI) analysis of spectral data. The following measures were used to control the quality of genotyping: (1) subjects with PTSD or without PTSD were mixed on each plate; (2) genotyping was performed blinded to the PTSD outcome of subjects; and (3) 10% of the samples were randomly selected for repeating genotyping, and the reproducibility was 100%. Consistent with previous studies focusing on trauma-related individuals [29, 44], those with one* (Met/Val)* or two* (Met/Met)* copies of the* Met* allele were combined together (*Met* carriers) and compared with* Val/Val* homozygotes.

### 2.5. Statistical Analysis

Statistical analyses were performed using SPSS version 19.0 (IBM Corp, Armonk, NY). All statistical tests were two-sided and were considered significant if the *P* value was less than 0.05. Descriptive statistics were analyzed and presented as frequencies (%) for categorical variables and means with corresponding standard deviations (SDs) for continuous variables. The Hardy-Weinberg equilibrium (HWE) for genotypic distribution was evaluated using the chi-square test for goodness of fit. The distribution of PTSD across sample characteristics and genotypes was assessed using the chi-square test, which was also used to assess the distribution of genotypes across sample characteristics.

The independent sample Student's *t*-test was used to compare the severity of PTSD symptoms between subjects with different genotypes. Univariable logistic regression analysis was used to assess the bivariate association between BDNF* Val66Met* polymorphism and PTSD, measured in terms of odds ratio (OR) with corresponding 95% confidence interval (95% CI). Multivariable logistic regression analysis was performed to explore the independent effect of BDNF Val66Met polymorphism on PTSD, measured in terms of adjusted OR (aOR) with corresponding 95% CI, adjusted for demographics, flood-related stressors, and postflood stressors.

## 3. Results

A total of 175 eligible subjects were enrolled in this study. All participants were Han ethnicity. Among all respondents, 94 (53.7%) were female, 152 (86.9%) were married, and 103 (58.9%) attended primary school at most. In addition, 74 (42.3%) and 45 (25.7%) experienced at least three flood-related stressors and at least three postflood stressors, respectively. The age for this study population ranged from 26 to 87 years with a mean (SD) age of 57.46 (11.48) years. According to the PCL-C cut-off score of 38, 28 individuals were diagnosed as having PTSD, indicating that the prevalence of PTSD among this study population at 17-year follow-up was 16.0% (28/175).

Table 1 displays the participants' characteristics according to PTSD status. Gender, flood-related stressors and postflood stressors were significantly associated with PTSD. Individuals with PTSD were more likely to be female (71.4% versus 50.3%, *P* < 0.05), experience at least three flood-related stressors (71.4% versus 36.7%, *P* < 0.05), and experience at least three postflood stressors (42.9% versus 22.4%, *P* < 0.05) than those without PTSD.

In addition, no significant deviation from the HWE was observed in the distribution of the genotypes (*χ*^2^ = 2.14, *P* = 0.342), with 40 (22.9%), 77 (44.0%), and 58 (33.1%) participants being* Val/Val* homozygous,* Val/Met* heterozygous, and* Met/Met* homozygous, respectively. Individuals with PTSD were more likely to carry the* Met* than those without it (92.9% versus 74.1%, *P* < 0.05).

Table 2 shows the participants' characteristics according to BDNF* Val66Met* carrier status. Among 135* Met* carriers, 73 (54.1%) were female, 59 (43.7%) experienced at least 3 flood-related stressors, and 33 (24.4%) experienced at least 3 postflood stressors. Participants' characteristics, namely, gender, age, marital status, education level, flood-related stressors, and postflood stressors, were not significantly associated with BDNF* Val66Met* carrier status (*P* > 0.05).

Table 3 presents the results of the logistic regression analyses of PTSD status. Univariable logistic regression analyses indicated that individuals with female gender (OR = 2.47, 95% CI = 1.02–5.95, *P* < 0.05), at least three flood-related stressors (OR = 4.31, 95% CI = 1.78–10.44, *P* < 0.05), at least postflood stressors (OR = 2.59, 95% CI = 1.12–6.02, *P* < 0.05), and* Met* allele (OR = 4.53, 95% CI = 1.03–20.01, *P* < 0.05) were more likely to develop PTSD than their counterparts. Multivariable logistic regression analysis indicated that female gender (aOR = 2.90, 95% CI = 1.04–8.08, *P* < 0.05), experiencing at least three flood-related stressors (aOR = 5.83, 95% CI = 2.18–15.59, *P* < 0.05), experiencing at least three postflood stressors (aOR = 2.85, 95% CI = 1.02–7.94, *P* < 0.05), and carrying the* Met* (aOR = 4.76, 95% CI = 1.02–22.15, *P* < 0.05) were risk factors for PTSD.

The mean (SD) PCL-C score for participants overall was 26.37 (9.12) and, for individuals with PTSD and without PTSD, the mean (SD) PCL-C scores were 40.29 (9.88) and 23.72 (6.07), respectively. When compared with the* Val/Val* homozygotes, the* Met* carriers had significantly higher PCL-scores. The mean (SD) PCL-C score for the* Val/Val* homozygotes was 23.60 (7.23), while, for the* Met* carriers, the mean (SD) PCL-C score was 27.19 (9.48) (Table 4).

## 4. Discussion

Despite a wide exploration of the association between BNDF* Val66Met* polymorphism and PTSD [26, 31], it has never been examined among flood survivors. To the best of our knowledge, this is the first study to explore the association of BNDF* Val66Met* polymorphism with the prevalence and severity of PTSD among flood survivors.

At 17-year follow-up, this study found that the prevalence of PTSD among flood survivors of the hard-hit areas of the 1998 Dongting Lake flood, who did not receive any psychological intervention, was 16.0%. This prevalence emphasizes the importance of early identification of risk factors and targeted psychological interventions for PTSD which might develop long time after a traumatic event [45]. Few studies have investigated PTSD beyond 17 years [46, 47]. Among those who did, one showed that 29% of Aberfan disaster survivors were diagnosed with PTSD at 33-year follow-up [46] and another found that 1.43% of Israeli Yom Kippur War veterans were still suffering from PTSD at 32-year follow-up [47]. Additional research, with shorter longitudinal follow-up times, has found, for example, PTSD prevalence of 9.7% among 9/11 police responders at 11–13-year follow-up [48] and PTSD prevalence of 9.2% among survivors five years after the 2008 Wenchuan earthquake in China [49]. The type of trauma exposure, the severity of exposure to the traumatic events, the follow-up time since the traumatic events, the characteristics of the study sample, and the instruments applied in the measurements may have contributed greatly to the different prevalence observed across studies [50, 51].

Since BDNF plays an important role in stress reaction and memory processing [19, 20], which were related to some clinical manifestations of PTSD, the role of BDNF in PTSD has been widely explored. As a functional SNP of the BDNF gene, which regulates activity-dependent secretion of BDNF from the neurons [24], BDNF* Val66Met* polymorphism has also been widely explored to examine its association with PTSD. In the current study, a significant association between BDNF* Val66Met* polymorphism and PTSD was observed. Specifically, this study found that* Met* carriers had more severe PTSD symptoms when compared with* Val/Val* homozygotes, which is consistent with a previous study by Li et al., who found that* Val* allele was associated with reduced severity of PTSD in male survivors of the Wenchuan earthquake [29].

Additionally, this study found that individuals with PTSD were more likely to be* Met* carriers than those without PTSD. Multivariable logistic regression analyses indicated that* Met* carriers were at higher risk for developing PTSD, when compared with* Val/Val* homozygotes. This is in line with a previous study, which indicated that the frequencies of* Val/Val* genotype were significantly higher in individuals without PTSD and those without the symptom of startle, than in individuals with PTSD and those with the symptom of startle [26].

The aetiology of PTSD is complex and the mechanism of the development of PTSD has not been fully understood [52]. Evidence has shown that individuals who carry the* Met* exhibited abnormal hippocampal activation [24], reduced hippocampal volumes [53], and impairment of learning and memory [54], which were frequently observed in PTSD patients [55]. This could, probably, explain why the current study found that* Met* carriers were more likely to develop PTSD than* Val/Val* homozygotes. Also, BDNF* Val66Met* polymorphism may result in differences in human brain serotonin system, thus affecting the risk for neuropsychiatric illness [56]. Besides, BDNF* Val66Met* polymorphism may affect BDNF serum levels thus implicated in the development of PTSD. Specifically, BDNF serum levels were lower in* Met* carriers than in* Val/Val* homozygotes [27, 28]. On the contrary, some studies found no association between this variant and BDNF serum levels [57, 58], while others found decreased BDNF serum levels in healthy* Val/Val* homozygotes when compared to* Val/Met* heterozygotes [59]. These inconsistent results across studies may be explained by a great variation in sample characteristics, assessment methods, and sample sizes. Therefore, additional studies with homogenous participants are needed to clarify the possible role of BDNF* Val66Met* polymorphism in PTSD.

Despite participants' characteristics not associated with BDNF* Val66Met* carrier status, in agreement with results from some previous studies [60, 61], this study found that females were more likely to develop PTSD and exhibit more severe PTSD symptoms when compared with males. For example, Skopp et al. found that the risk for PTSD among 2,583 soldiers following Iraq deployment was nearly 2.5 times greater for women, as compared to men [61]. Gender difference in PTSD could be largely explained by gender difference in interpretation of the traumatic event, as well as gender difference in coping strategies when in distress [62]. In particular, females interpret negative events much more stressfully than their male counterparts [62].

In addition, flood-related stressors and postflood stressors were found to be associated with PTSD in this study, and this is consistent with results from many previous studies. Zhang et al. found that individuals who lost family members due to the 2008 Wenchuan earthquake were more likely to develop PTSD after the earthquake [49], and Cone et al. found that having more than two injuries due to the 9/11 attacks was a risk factor for chronic probable PTSD among police responders [63]. Furthermore, Cone et al. observed that experiencing more than two stressors in the last 12 months and more than two life-threatening events since 9/11 was associated with an increased risk of developing chronic probable PTSD among police responders [63]. Similarly, the results of this study indicated that experiencing at least three postflood stressors increased the likelihood of developing PTSD long time after the flood, which strongly support the view that stressful events impact significantly on long-term progress of PTSD [64].

When interpreting the findings of this study, it is worth noting here that participants were enrolled in this study 17 years after they had experienced the flood disaster. Many survivors may be lost at 17-year follow-up because of death or migration. Thus, those who enrolled in this study may have different characteristics from the unreachable individuals, which may cause potential selection bias. Due to the cross-sectional study design, baseline data were not available in this study. Therefore, it was unable to compare the characteristics of those who were enrolled in this study with those unreachable. Besides, albeit significant, the 95% CI of the odds ratio for the* Met* carriers' risk of PTSD versus* Val/Val* homozygotes' was wide, suggesting that the statistical power was relatively low when exploring the association between BDNF* Val66Met* polymorphism and PTSD. Furthermore, its lower limit was 1.02, almost overlapping the null value. This was mainly due to the limited sample size of this study. Future longitudinal studies with large sample size are warranted to detect a reliable and precise estimation.

Certain limitations should be acknowledged in this study. First, PTSD status in this study was identified using self-report screening questionnaire, instead of formal clinical interview. Although the PCL-C has been widely used with high psychometric properties when structured clinical interview was not feasible, it is essentially a screening tool. As a consequence, the prevalence of PTSD found in this study may be overestimated. Second, although some previous studies have shown that there may be interactions between BDNF* Val66Met* polymorphism and some environmental factors such as age, gender, and traumatic load [26, 29], stratified analyses according to age, gender, flood-related stressors, or postflood stressors were not performed in this study due to the limited sample size. However, the finding that BDNF* Val66Met* carrier status was not associated with participants' characteristics, including gender, age, marital status, education level, flood-related stressors, and postflood stressors, indicated that the probability of interactions between BDNF* Val66Met* carrier status and the preceding factors might be low. Third, BDNF peripheral levels were not explored in this study. Fourth, this cross-sectional study was conducted 17 years after the 1998 Dongting Lake flood. Thus, recall bias could exist when participants responded to questions about flood-related stressors and postflood stressors. However, the results that flood-related and postflood stressors significantly associated with PTSD in this study were in accordance with results from many previous studies [63, 64]. Finally, participants who were enrolled in this study were all Chinese of Han ethnicity. Whether findings from this study could be generalized into other populations needs to be replicated.

## 5. Conclusions

This cross-sectional study found that 16.0% of the survivors who experienced the 1998 Dongting Lake flood still suffered from PTSD at 17-year follow-up. Survivors with PTSD were more likely to be female, experience at least three flood-related stressors, experience at least three postflood stressors, and carry the* Met*. Compared with* Val/Val* homozygotes,* Met* carriers for BDNF rs6265 were at higher risk of developing PTSD and also exhibited more severe PTSD symptoms.

## Figures and Tables

**Table 1:** Participants' characteristics by PTSD status.

Variable	Category	Total *n* (%)	PTSD status		*χ* ^2^ value	*P* value
			Yes *n* (%)	No *n* (%)		
Overall		175 (100.0)	28 (16.0)	147 (84.0)		
Gender	Female	94 (53.7)	20 (71.4)	74 (50.3)	4.207	0.040^*∗*^
	Male	81 (46.3)	8 (28.6)	73 (49.7)		
Age (years)	29–59	103 (58.9)	18 (64.3)	85 (57.8)	0.406	0.524
	≥60	72 (41.1)	10 (35.7)	62 (42.2)		
Marital status	Married	152 (86.9)	25 (89.3)	127 (86.4)	0.172	0.678
	Single/divorced/widowed	23 (13.1)	3 (10.7)	20 (13.6)		
Education level	≤primary school	103 (58.9)	13 (46.4)	90 (61.2)	2.126	0.145
	>primary school	72 (41.1)	15 (53.6)	57 (38.8)		
Flood-related stressors	0–2	101 (57.7)	8 (28.6)	93 (63.3)	11.600	0.001^*∗*^
	≥3	74 (42.3)	20 (71.4)	54 (36.7)		
Postflood stressors	0–2	130 (74.3)	16 (57.1)	114 (77.6)	5.128	0.024^*∗*^
	≥3	45 (25.7)	12 (42.9)	33 (22.4)		
BDNF *Val66Met* carrier status	*Val/Val*	40 (22.9)	2 (7.1)	38 (25.9)	4.668	0.031^*∗*^
	*Met/Met* + *Met/Val*	135 (77.1)	26 (92.9)	109 (74.1)		

^*∗*^ *P* < 0.05.

**Table 2:** Participants' characteristics by BDNF *Val66Met* carrier status.

Variable	Category	Total *n* (%)	BDNF *Val66Met* carrier status		*χ* ^2^ value	*P* value
			*Val/Val* *n* (%)	*Met/Met* + *Met/Val* *n* (%)		
Overall		175 (100.0)	40 (22.9)	135 (77.1)		
Gender	Female	94 (53.7)	21 (52.5)	73 (54.1)	0.031	0.861
	Male	81 (46.3)	19 (47.5)	62 (45.9)		
Age (years)	29–59	103 (58.9)	24 (60.0)	79 (58.5)	0.028	0.867
	≥60	72 (41.1)	16 (40.0)	56 (41.5)		
Marital status	Married	152 (86.9)	38 (95.0)	114 (84.4)	3.102	0.083
	Single/divorced/widowed	23 (13.1)	2 (5.0)	21 (15.6)		
Education level	≤primary school	103 (58.9)	21 (52.5)	82 (60.7)	0.865	0.352
	>primary school	72 (41.1)	19 (47.5)	53 (39.3)		
Flood-related stressors	0–2	101 (57.7)	25 (62.5)	76 (56.3)	0.487	0.485
	≥3	74 (42.3)	15 (37.5)	59 (43.7)		
Postflood stressors	0–2	130 (74.3)	28 (70.0)	102 (75.6)	0.499	0.480
	≥3	45 (25.7)	12 (30.0)	33 (24.4)		

**Table 3:** Logistic regression analyses of the effects of participants' characteristics and BDNF *Val66Met* polymorphism on PTSD status.

Variable	Category	Univariable analyses		Multivariable analyses	
		OR (95% CI)	*P* value	aOR (95% CI)	*P* value
Gender	Male	1		1	
	Female	2.47 (1.02–5.95)	0.045^*∗*^	2.90 (1.04–8.08)	0.042^*∗*^
Age (years)	29–59	1		1	
	≥60	0.76 (0.33–1.76)	0.525	1.32 (0.44–3.94)	0.615
Marital status	Married	1		1	
	Single/divorced/widowed	0.762 (0.21–2.76)	0.679	0.44 (0.10–1.88)	0.264
Education level	>primary school	1		1	
	≤primary school	0.549 (0.24–1.24)	0.148	0.51 (0.19–1.36)	0.177
Flood-related stressors	0–2	1		1	
	≥3	4.31 (1.78–10.44)	0.001^*∗*^	5.83 (2.18–15.59)	<0.001^*∗*^
Postflood stressors	0–2	1		1	
	≥3	2.59 (1.12–6.02)	0.027^*∗*^	2.85 (1.02–7.94)	0.045^*∗*^
BDNF *Val66Met* carrier status	*Val/Val*	1		1	
	*Met/Met* + *Met/Val*	4.53 (1.03–20.01)	0.046^*∗*^	4.76 (1.02–22.15)	0.047^*∗*^

^*∗*^ *P* < 0.05.

**Table 4:** PCL-C score (Mean ± SD) by BDNF *Val66Met* carrier status.

	BDNF *Val66Met* carrier status		*t* value	*P* value
	*Val/Val*	*Met/Met* + *Met/Val*		
PCL-C score	23.60 ± 7.23	27.19 ± 9.48	−2.213	0.028^*∗*^

^*∗*^ *P* < 0.05.
